# School and Industrial Hygiene

**Published:** 1880-10

**Authors:** 


*School and Industrial Hygiene.*—By D. F. Lincoln, M. D. Publisher: Presley Blakiston, Philadelphia, 1880.

Another of the American Health Primers to which the phrase *multum in parvo* can be appropriately applied. Parents and teachers are directly interested in this subject and will find a great deal of useful information given in this small treatise. Its contents consist of Articles on Emotional and Mental Strain, Food and Sleep, Bodily Growth, Amount of Study, Exercise, Care of the Eyes, School Desks and Seats, A Model School Room, Ventilation and Heating, Site, Drainage, etc., Private Schools, Colleges, Contagious Diseases, Injurious Effects of Inhaling Dust and Poisonous Substances, Injuries from Atmospheric Changes, Injuries from Over-use of Certain Organs, Injuries from Accidents, Duration of Life in Various Occupations.

---

